# ATP prevents Woronin bodies from sealing septal pores in unwounded cells of the fungus Zymoseptoria tritici

**DOI:** 10.1111/cmi.12764

**Published:** 2017-08-09

**Authors:** Gero Steinberg, Martin Schuster, Christian Hacker, Sreedhar Kilaru, Ana Correia

**Affiliations:** ^1^ School of Biosciences University of Exeter Exeter UK; ^2^ Bioimaging Centre University of Exeter Exeter UK; ^3^ Department of Biology University of Utrecht Utrecht The Netherlands

**Keywords:** cell rupture, Hex1, septal pore, Septoria tritici wheat blotch, Woronin body, Zymoseptoria tritici

## Abstract

Septa of filamentous ascomycetes are perforated by septal pores that allow communication between individual hyphal compartments. Upon injury, septal pores are plugged rapidly by Woronin bodies (WBs), thereby preventing extensive cytoplasmic bleeding. The mechanism by which WBs translocate into the pore is not known, but it has been suggested that wound‐induced cytoplasmic bleeding “flushes” WBs into the septal opening. Alternatively, contraction of septum‐associated tethering proteins may pull WBs into the septal pore. Here, we investigate WB dynamics in the wheat pathogen Zymoseptoria tritici. Ultrastructural studies showed that 3.4 ± 0.2 WBs reside on each side of a septum and that single WBs of 128.5 ± 3.6 nm in diameter seal the septal pore (41 ± 1.5 nm). Live cell imaging of green fluorescent ZtHex1, a major protein in WBs, and the integral plasma membrane protein ZtSso1 confirms WB translocation into the septal pore. This was associated with the occasional formation of a plasma membrane “balloon,” extruding into the dead cell, suggesting that the plasma membrane rapidly seals the wounded septal pore wound. Minor amounts of fluorescent ZtHex1‐enhanced green fluorescent protein (eGFP) appeared associated with the “ballooning” plasma membrane, indicating that cytoplasmic ZtHex1‐eGFP is recruited to the extending plasma membrane. Surprisingly, in ~15% of all cases, WBs moved from the ruptured cell into the septal pore. This translocation against the cytoplasmic flow suggests that an active mechanism drives WB plugging. Indeed, treatment of unwounded and intact cells with the respiration inhibitor carbonyl cyanide m‐chlorophenyl hydrazone induced WB translocation into the pores. Moreover, carbonyl cyanide m‐chlorophenyl hydrazone treatment recruited cytoplasmic ZtHex1‐eGFP to the lateral plasma membrane of the cells. Thus, keeping the WBs out of the septal pores, in Z. tritici, is an ATP‐dependent process.

**Abbreviations**
CCCP: carbonyl cyanide *m*‐chlorophenyl hydrazone
eGFP: enhanced green fluorescent protein
WB: Woronin body

## 1 INTRODUCTION

The Pezizomycetes are the largest class within the ascomycetes. This fungal group includes important human and plant pathogens, such as *Aspergillus fumigatus* and *Zymoseptoria tritici*, the causal agent of *Septoria tritici* blotch in wheat. Pezizomycetes invade their substrates by tip growing multicellular hyphae, in which cells are separated by septa. These septa are perforated by a septal pore that ensures communication and exchange of cytoplasm and organelles (overview in Steinberg, Peñalva, Riquelme, Wösten, & Harris, 2017), required for fungal development and radial colony growth (Trinci, 1973). However, this hyphal architecture bears the risk that wounding of individual cells causes extensive cytoplasmic bleeding and catastrophic damage to the entire hypha. To meet this challenge, the Pezizomycetes have developed an efficient protection mechanism, on the basis of the rapid closure of septal pores by Woronin bodies (WBs; Jedd & Pieuchot, 2012). These peroxisome‐derived spherical organelles were first described as septum‐associated refractive particles in *Ascobolus pulcherrimus* (Woronin, 1865) and subsequently found in numerous fungi (overview in Markham & Collinge, 1987). WBs are usually associated with the pore on both sides of the septum, although cytoplasmic WBs have also been described (Beck, Echtenacher, & Ebel, 2013; Momany, Richardson, Van Sickle, & Jedd, 2002). Early ultrastructural reports implied WBs in damage‐induced sealing of septal pores (Reichle & Alexander, 1965; Trinci & Collinge, 1974). Moreover, null mutants in *hex1*, a gene encoding a major WB protein discovered in *Neurospora* crassa (Jedd & Chua, 2000; Tenney et al., 2000), lack WBs and are unable to rescue their hyphal cells upon damage (Beck et al., 2013; Han, Jin, Maruyama, & Kitamoto, 2014; Jedd & Chua, 2000; Maruyama, Juvvadi, Ishi, & Kitamoto, 2005). These findings strongly argue that WBs “guard” the septal pores to seal off damaged cells and so limit the damage to the hypha.

The mechanism by which WBs plug the septal pore is not understood. The most widely accepted hypothesis is that wound‐induced bulk flow of cytoplasm “flushes” septum‐associated WBs from the unwounded cell into the septal pore (Jedd & Chua, 2000; Markham & Collinge, 1987; Maruyama et al., 2005). However, quantitative electron microscopy studies revealed that a single WB closes the septum after wounding, whereas other WBs remain largely unaffected. This was taken as an argument against a pressure‐driven mechanism of pore sealing by WBs (Markham & Collinge, 1987). Alternatively, a contractile tether may pull WBs into the septal pore (Markham & Collinge, 1987). This hypothesis is supported by optical laser trapping experiments in *Nectria haematococca*, which revealed “elastic” tethering of WBs to the septal pore (Berns, Aist, Wright, & Liang, 1992). Indeed, studies in N. crassa identified the protein LAH1 as being such tether (Ng, Liu, Lai, Low, & Jedd, 2009), and its homologue in A. fumigatus and *Aspergillus oryzae* was shown to anchor WBs at the septal pore (Beck et al., 2013; Han et al., 2014; Leonhardt, Carina Kakoschke, Wagener, & Ebel, 2017). Lah‐homologues share sequence similarity to motifs in the muscle protein titin (Ng et al., 2009), which confer calcium‐dependent elasticity to titin (Labeit et al., 2003). With this finding, controlled contraction of Lah was suggested to mediate WB plugging (Han et al., 2014). However, no experimental evidence for such a mechanism exists. Interestingly, mutant studies in N. crassa strongly suggest a role of the septum‐associated protein SPA9 in preventing Woronin‐based septal pore plugging (Lai et al., 2012). The molecular mechanism behind this is not known, but this finding adds strong support to the notion that WB‐based pore plugging is an active process.

In this study, we use electron microscopy and live cell imaging to elucidate WB dynamics after laser‐based hyphal wounding in *Z. tritici*. This fungus causes Septoria wheat blotch and poses a serious challenge to wheat producing agricultural industry (Fones & Gurr, 2015). However, despite its economic importance, its cell biology is poorly understood (Steinberg, 2015). We show that cell injury creates a pressure gradient, which is consistent with cytoplasmic flow‐driven movement of WBs into the septal pore. However, a subpopulation of the WBs moves against the flow from the ruptured cell into the septal pore, suggesting an active mechanism of WB‐based pore plugging. In agreement with this notion, we report that reduced cellular ATP levels trigger movement of WBs into the septal pore in intact hyphae.

## 2 RESULTS

### 2.1 A large number of Woronin bodies “guard” the septal pore

**Figure 1:**
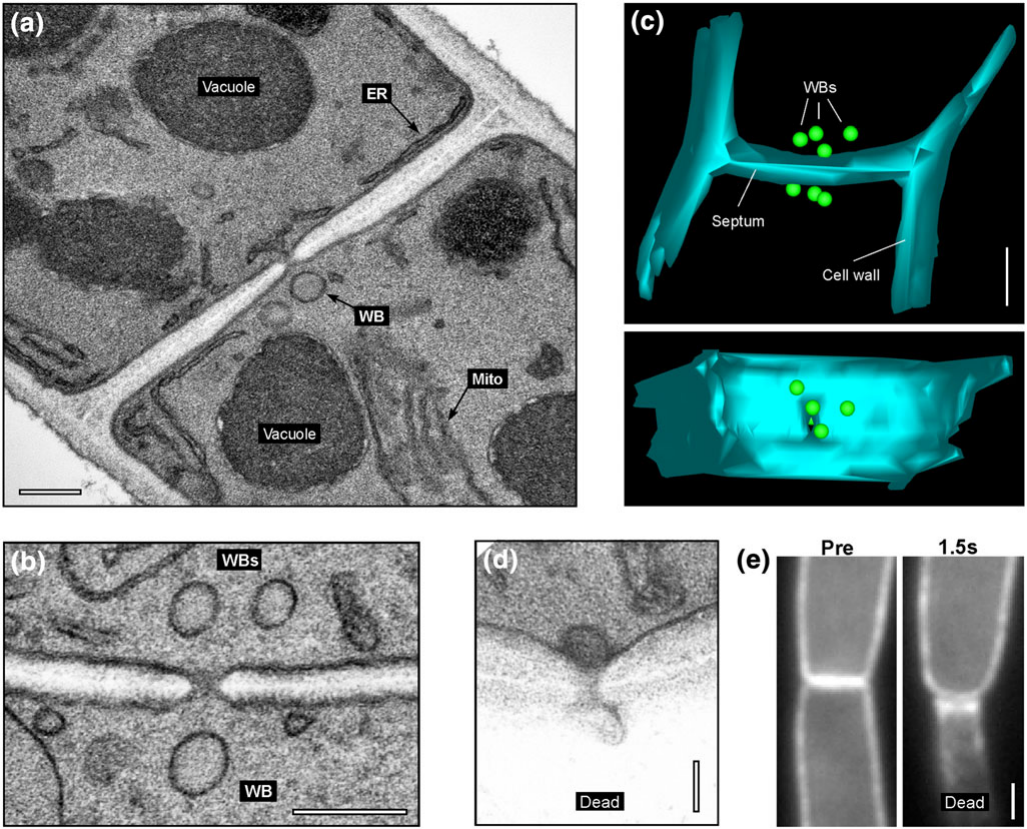
Woronin bodies in *Zymoseptoria tritici*. (a) Electron micrograph showing a septum in *Z. tritici*. A single Woronin body (WB) is indicated. Scale bar represents 0.2 μm. (b) Electron micrograph showing a septum in *Z. tritici*. Several WBs surround the septal pore. Scale bar represents 0.2 μm. (c) A 3D reconstruction of serial sections through a septum of *Z. tritici*. Scale bar represents 0.5 μm. See also supplementary Movie **S1**. (d) Electron micrograph of the septal pore of a wild‐type cell of strain IPO323 after wounding with quartz sand. A single WB has sealed the septal pore on the side of the intact cell. The injured cell has collapsed (dead cell). Scale bar represents 0.1 μm. (e) Behaviour of a septum, labelled with the plasma membrane marker eGFP‐Sso1, after laser‐induced rupture of the lower cell (indicated by “Dead”). The septum bends towards the collapsed cell, indicating a pressure gradient. Scale bar represents 1 μm. See also Movie **S4**

As a first step in our study, we set out to analyse WB localisation, number, and dimension in the *Z. tritici* wild‐type strain IPO323, using electron microscopy techniques in chemically fixed cells. Consistent with reports in other fungi, spherical WBs were closely associated with the septal pore (Figure 1a, 1b). These rounded organelles were surrounded by a single membrane and displayed a fine‐granular homogeneous matrix. They had a diameter of ~129 nm, whereas the septal pore opened only ~41 nm and were located at average ~300 nm away from the pore (Table 1). To determine the number of septum‐associated WBs, we generated image stacks, derived from 24 to 26 serial sections per septum. Using this 3D information, we determined that three to four WBs “guard” each side of the septal pore in *Z. tritici* (Table 1; Figure 1c, Movie **S1**). Next, we treated cells of *Z. tritici* with quartz sand crystals and visualised septal pores in these wounded cells. We found that septa were always plugged by a single WB (*n* = 20; Figure 1d). The remaining two to three WBs in the intact cell only slightly changed their position relative to the plugged septal pore (average distance to septal pore: 284.02 ± 21.22 nm, *n* = 45; mean ± standard error of the mean; not significantly different from control, *p* = 0.6978).

**Table 1:** Woronin bodies in Zymoseptoria tritici

WB diameter (nm)^a^	Septal pore opening (nm)	Number of septal WBs per cell	Number of cytopl. WBs per cell^b^	Distance of WBs to septal pore^a^ (nm)	WBs in pore^c^ (%)
128.5 ± 3.6 (50)	41.0 ± 1.7 (34)	3.4 ± 0.2 (20)	6.0 ± 0.8 (25)	299.0 ± 32.1 (73)	7.65 ± 7.05 (3 exp.)

*Note*. All values given as mean ± standard error of the mean (sample size).

eGFP = enhanced green fluorescent protein; WB = Woronin body.

a Dimensions of septum‐associated WBs.

b Cytoplasmic WBs are defined as clearly visible signals of ZtHex1‐eGFP.

c A single strong ZtHex1‐eGFP in the centre of the septum expressing cells; 251 septa from 3 experiments were analysed.

### 2.2 Woronin bodies plug the pore against high pressure

Cell injury induces cytoplasmic bleeding. This, in turn, may “flush” WBs from neighbouring intact cells into the septal pore (Jedd & Chua, 2000; Markham & Collinge, 1987; Maruyama et al., 2005). We investigated cytoplasmic bleeding by performing controlled cell wounding experiments, using a 405‐nm laser pulse to rupture cells. We did this in cells that express cytoplasmic green fluorescent protein (GFP), or the plasma membrane marker enhanced GFP (eGFP)‐ZtSso1, and observed the effect of wounding on cytoplasmic bleeding and on the septa, using live cell imaging. We found that wounding induced bleeding of the GFP‐containing cytoplasm from the wounded cell into the extracellular space (Movie **S2**). Rapid sealing by the pore prevented damage to the neighbouring cell (Movie **S3**; observed in all of the 69 laser‐rupture experiments), and bending of the sealed septum induced a significant pressure difference between the intact and injured cell compartments (Figure 1e, Movie **S4**). This indicates that rapid plugging prevents further damage in adjacent cells.

**Figure 2:**
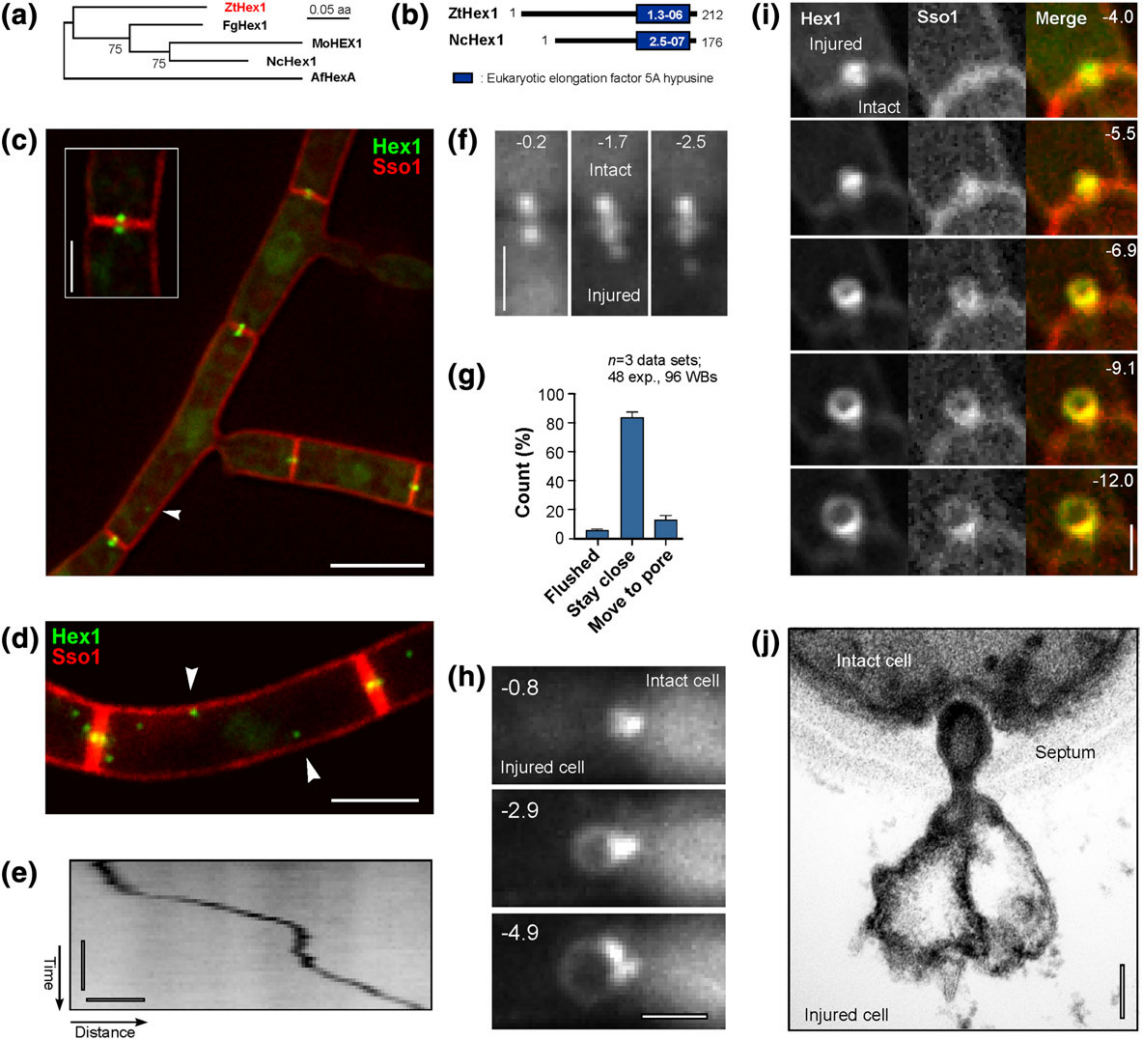
Identification and live cell imaging of ZtHex1‐GFP. (a) Phylogenetic tree comparing the predicted amino acid sequence of fungal homologues of ZtHex1. NCBI accession numbers are as follows: *Zymoseptoria tritici* ZtHex1, XP 003854425.1; *Magnaporthe oryzae* MoHEX1, XP 003721069.1; *Neurospora crassa* NcHex1, EAA34471.1; *Fusarium graminearum* FgHex1, SCB65655.1; *Aspergillus fumigatus* AfHex, KMK59524.1. Maximum‐likelihood trees were generated using MEGA5.2. Bootstrap values from 500 rounds of calculation are indicated at branching points. Tree was generated in MEGA5.2; http://www.megasoftware.net/. (b) Comparison of the predicted domain structure of ZtHex1 from *Z. tritici* and NcHex1 from N. crassa. Error probabilities were determined in PFAM and are given in white numbers. (c) *Z. tritici* cells, coexpressing the Woronin body (WB) marker ZtHex1‐GFP and the red fluorescent plasma membrane protein mCherry‐Sso1. Strong ZtHex1‐GFP signals are concentrated on both sides of the septum (inset). In addition, nonmotile WBs of weaker fluorescent intensity locate in the cytoplasm (arrowhead). Scale bar represents 5 μm. (d) Maximum projection of a *z*‐axis stack of images, showing numerous cytoplasmic WBs (arrowhead). Scale bar represents 3 μm. (e) Contrast‐inverted kymograph showing directed motility of a cytoplasmic WB. Horizontal bar represents 2 s; vertical bar represents 1 μm. See also Movie **S5**. (f) WB behaviour after laser wounding of *Z. tritici* cells. Immediately after injury, the cellular pressure drops in the wounded cell (lower half of images, indicated by “Dead”). The WB of the intact cell has plugged the septal pore, whereas WBs in the wounded cell remain stationary or move slightly away from the septum, whereas the cytoplasm bleeds out. Time after wounding is given in seconds. Scale bar represents 1 μm. See also Movies **S6** and **S7**. (g) Bar chart showing the behaviour of WBs in laser‐wounded cells. In most cases, the WBs in the ruptured cell stay associated with the septum. Mean ± standard error of the mean is shown; sample size *n* is 3 data sets, 48 experiments. (h) WB “ballooning” in a laser‐wounded cell of *Z. tritici*. After injury of the cell (left half of images, indicated by “Dead”), the WB of the intact cell plugs the pore. Within a few seconds, the WB balloons out, whereas the cytoplasm bleeds out of the ruptured cell. Time after wounding is given in seconds; scale bar represents 1 μm. See also Movie **S8**. (i) Image series showing “ballooning” of ZtHex1‐eGFP and mCherry‐ZtSso1 after injury of a cell. The “balloon” contains the integral syntaxin ZtSso1, suggesting that the plasma membrane in the unwounded cell (indicated by “intact”) sealed after wounding and extends due to the pressure gradient into the wounded cell (indicated by “injured”). Time in seconds given in the upper right corner; scale bar represents 1 μm. See also Movie **S9**. (j) Electron micrograph of the septal pore of a wild‐type cell after wounding with quartz sand. A WB has sealed the septal pore and formed a “balloon” into the injured cell (dead cell). Note that the membrane of the WB extends into the “bubble,” suggesting that the pressure gradient between the injured cell (dead cell) and the living cell (live cell) causes shape change of the organelle. Scale bar represents 0.1 μm

Hex1 encodes the major protein in the hexagonal crystals in WBs in filamentous ascomycetes. It was used to visualise WBs in living fungal cells (overview in Steinberg et al., 2017). We used the predicted amino acid sequence of Hex1 from N. crassa and identified a putative homologue, ZtHex1, in the published genomic sequence of *Z. tritici* (Goodwin et al., 2011). ZtHex1 shares 59.7% amino acid sequence identity with Hex1 in N. crassa, and it groups with other Hex1‐like protein orthologues in a maximum‐likelihood tree (Figure 2a). In addition, ZtHex1 shares a eukaryotic elongation factor 5A hypusine domain (eIF5A domain) with NcHex1 from N. crassa (Figure 2b). Taken together, these results leave little doubt that ZtHex1 is a WB‐associated Hex1‐like protein, involved in WB‐based sealing of the septal pore.

To visualise WBs in living cells, we fused *hex1* to the *egfp* gene, encoding eGFP. We coexpressed the fusion protein (ZtHex1‐eGFP) with the plasma membrane marker mCherry‐ZtSso1 (Kilaru, Schuster, Ma, & Steinberg, 2017). Consistent with our ultrastructural results, a pair of strong ZtHex1‐eGFP signals was located next to the septa in the multicellular *Z. tritici* structures (Figure 2c) in 92.4 ± 7.1 of all cells (*n* = 3 experiments, 72–90 septa per experiment analysed; Table 1). In the remaining 7.6%, a single ZtHex1‐eGFP “dot” was located in the septal pore region, indicating that the septum was closed by WBs in a small number of cells. It is worth mentioning that the strong ZtHex1‐eGFP signals most likely represent numerous septa‐associated WBs, which cannot be separated spatially by light microscopy. Moreover, the cytoplasm contained additional weaker ZtHex1‐GFP signals, which may represent a population of cytoplasmic WBs (arrowheads Figure 2d, maximum projection of a *z*‐axis image stack; Table 1), previously described in *Aspergillus* nidulans and A. fumigatus (Beck & Ebel, 2013; Momany et al., 2002). The majority of these cytoplasmic WBs were nonmotile, with rapid directed movement only rarely visible (Figure 2e, contrast‐inverted kymograph shows motility as a diagonal line; Movie **S5**).

We considered it likely that WBs are responsible for the wound‐induced rapid plugging of the septal pore. To test this, we observed ZtHex1‐eGFP and mCherry‐ZtSso1 in laser‐induced rupture experiments. Indeed, in all experiments (*n* = 58), WBs moved into mCherry‐ZtSso1‐labelled septum (Movie **S6**). In most cases, the WBs in the ruptured cell remained associated with the septum (Figure 2f, 2g; Movie **S7**), and only in 5.2% of all experiments did cytoplasmic bleeding wash the WBs out of the ruptured cell (Figure 2g). Occasionally, plugging by WBs was accompanied by dynamic rearrangement of ZtHex1‐eGFP in the region of the septal pore (Movie **S8**, #1 and #3) or by the formation of ZtHex1‐eGFP “balloons” (Figure 2h; Movie **S8**, #2 and #4). Co‐observation of ZtHex1‐eGFP and mCherry‐ZtSso1 revealed that these “balloons” are derived from the plasma membrane (Figure 2i; Movie **S9**). Electron microscopy confirmed that the “balloons” were surrounded by a double membrane and contain peripheral granular material (Figure 2j). This may represent ZtHex1‐eGFP, visible as a GFP lining of the expanding plasma membrane “balloon” (Movies **S8** and **S9**). The origin of this ZtHex1‐eGFP lining is not known, but it appears likely that it is recruited from a cytoplasmic pool of this protein. The physiological relevance of such cytoplasmic ZtHex1‐eGFP, however, is not known, but “ballooning” of mCherry‐ZtSso1 suggests rapid sealing of the plasma membrane after rupture of the cell, which is too weak to resist the pressure gradient between the intact and bleeding cells.

### 2.3 An active mechanism participates in Woronin body closure of septal pores

**Figure 3:**
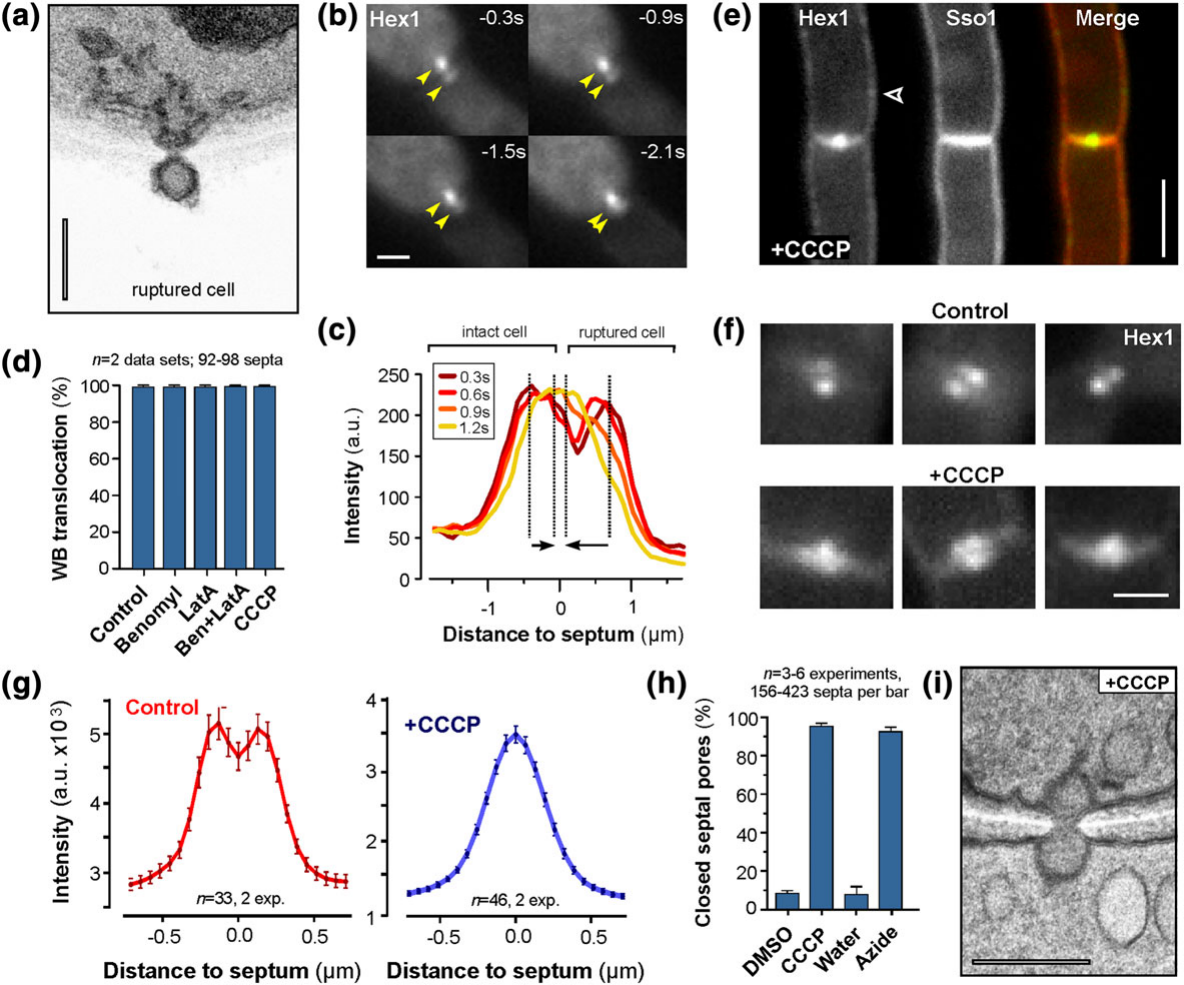
ATP‐dependent Woronin body (WB) closure of septal pores. (a) Electron micrograph showing plugged septal pores after wounding with quartz sand of the lower cell (ruptured cell). A single WB seals the pore in the dead compartment. Scale bar represents 0.2 μm. (b) Image series showing recruitment of WBs from the ruptured cell (dead) into the septum. Yellow arrowheads indicate fusion of the ZtHex1‐GFP signals in the septal pore. Time after wounding is given in seconds; scale bar indicates 1 μm. See also Movie **S10**. (c) Graph showing fluorescent intensity scan curves of a pair of WBs over time. At 1.2 s after cell wounding, the WBs from the ruptured cell (left), as well as the WBs from the intact cell (right), have moved towards the septum and their fluorescent maximum overlaps. Arrows indicate the displacement of the intensity maxima over the 900‐ms observation time; time in seconds after wounding is indicated in box and colour coded. (d) Bar chart showing the number of septa that are sealed after laser rupture and in the presence of the microtubule inhibitor benomyl (Ben), the F‐actin inhibitor latrunculin A (LatA), and an inhibitor of oxidative phosphorylation (CCCP). Mean ± standard error of the mean is shown; sample size *n* is 2 data sets, 92–98 septa per bar, observed in >50 shooting experiments. (e) *Zymoseptoria tritici* cells expressing mCherry‐ZtSso1 and ZtHex1‐GFP that were treated with 100 μM CCCP for 15 min. The two septum‐associated WB signals fuse into one signal, which is located in the septal pore. In addition, small amounts of ZtHex1‐eGFP were found at the plasma membrane (arrowhead). Scale bar represents 3 μm. (f) Examples showing septum‐associated WBs in untreated control cells (upper gallery) and in CCCP‐treated cells (lower gallery). Depletion of cellular ATP concentrates WBs in the septal pore. Scale bar represents 1 μm. (g) Graph showing intensity curves over WBs at septa of control cells and after treatment with 100 μM CCCP for 15 min. In the presence of the respiration inhibitor, the bimodal distribution of ZtHex1‐GFP turns into a unimodal distribution, indicating that the WBs have moved into the septal pore. Note that the cells were not injured. Also note that CCCP treatment reduces the overall ZtHex1‐GFP fluorescent intensity in the cell. (h) Bar chart showing the number of septa that are sealed after treatment of 10–15 min with 100 μM CCCP or 0.1% sodium azide in unwounded multicellular structures. Mean ± standard error of the mean is shown; sample size *n* is 3–6 data sets, with 156–423 septa analysed per bar. (i) Electron micrograph showing WBs at septal pores in CCCP‐treated cells (+CCCP). Reducing the ATP levels results in WBs plugging on both sides of the septum. Scale bar represents 0.2 μm

Our ultrastructural analysis also revealed that some pores were sealed by WBs from the ruptured cell side (Figure 3a; Figure **S1**; seen in 3 out of 20 cases, equals ~15%). Indeed, live cell imaging of ZtHex1‐eGFP in cell wounding experiments confirmed that WBs are able to move from the ruptured cell into the septal pore (seen in 12.3% of all experiments, *n* = 3 data sets; Figure 3b, “Move to pore”; Movie **S10**). This movement is best seen in fluorescent intensity scans over the pair of ZtHex1‐eGFP signals. Here, the two fluorescent intensity peaks, representing the WBs in the intact and ruptured cells, form one intensity peak within 1–2 s after wounding (Figure 3c). Considering the wound‐induced drop in pressure, this WB motion occurs against the cytoplasmic bleeding. This suggests the existence of an active mechanism of WB movement into the septal pores.

### 2.4 Woronin body plugging of pores requires cellular ATP

The cytoskeleton has been implied in septal pore plugging by WBs (Markham & Collinge, 1987). To test for a role of the cytoskeleton in pore closure, we observed ZtHex1‐eGFP in laser‐injured cells that were treated with benomyl and latrunculin A. These inhibitors have been shown to disassemble microtubules and F‐actin in *Z. tritici* (Kilaru et al., 2017; Schuster, Kilaru, Latz, & Steinberg, 2015). Upon cell wounding, WBs moved into the septal pore in control cells, as well as in cells treated with benomyl and latrunculin A (Figure 3d). This result suggests that the cytoskeleton is not involved in WB‐mediated pore plugging. To test if ATP‐dependent enzymatic activity is required to close the septal pore, we treated the cells with the carbonyl cyanide *m*‐chlorophenyl hydrazone (CCCP). This chemical inhibitor of oxidative phosphorylation reversibly depletes cellular ATP in fungal cells (Lin et al., 2016). We found that CCCP‐treated cells were not impaired in plugging the septal pore (Figure 3d), suggesting that moving WBs into the pore does not involve enzymatic activity. However, in the presence of CCCP, even unwounded cells moved their WBs into the septal pore, resulting in single ZtHex1‐eGFP signals in the middle of the septum (Figure 3e, 3f, 3g). In addition, small amounts of ZtHex1‐eGFP were recruited to the plasma membrane (Figure 3e, arrowhead), again suggesting the existence of a cytoplasmic pool of ZtHex1. Quantitative analysis, using linescan intensity scans over the septal pore, showed that 10 min CCCP treatment resulted in WBs sealing of 95.8 ± 3.6% of all septa, indicated by a single strong fluorescent signal in the centre of the septum (*n* = 6 experiments, 51–83 septa analysed per experiment). In contrast, treatment with the solvent dimethyl sulfoxide alone has no effect on septal pore closure (7.8 ± 4.2% of the pores are closed, *n* = 6 experiments, 52–94 septa analysed per experiment; Figure 3h; no significant difference to untreated cells, *p* = .9792; Student's *t* test). Ultrastructural studies confirmed that two WBs plugged the septal pores in these intact but CCCP‐treated cells (Figure 3i). Finally, we treated cells with sodium azide, a drug that accumulates in mitochondria as an anion and that inhibits the mitochondrial respiration (Palmieri & Klingenberg, 1967). Consistent with a role of ATP in WB translocation, the azide‐induced inhibition of ATP synthesis shifted ZtHex1‐eGFP‐labelled WBs into the septal pore in unwounded cells (Figure 3h). Taken together, these results strongly suggest that ATP is required to prevent WBs from sealing the septal pore open.

## 3 DISCUSSION

Cell–cell communication through septal pores ensures long‐range communication and exchange of cytoplasm, proteins, and organelles throughout the length of the hypha (overview in Steinberg et al., 2017). Local injury and subsequent cytoplasmic bleeding pose a challenge to such interconnected system of cells, as it bears the risk of catastrophic damage to the entire cell chain. WB‐based plugging of the septal pore is an efficient mechanism to protect the fungal hypha. However, despite intensive research, the actual mechanism by which WBs are translocated from the cytoplasm into the septal pore remains elusive. Three fundamentally different mechanisms have been suggested: (a) passive “flushing in” of WBs by cytoplasmic bulk flow from the intact to the wounded cell, (b) active transport of WBs to the pore along the cytoskeleton, and (c) active contraction of an elastic tether that drags WBs towards the septal pore (Markham & Collinge, 1987). We discuss our results in the light of these proposed mechanisms.

Hyphal cells build up internal turgor pressure (Lew, 2011), which, upon wounding, causes bleeding of the cytoplasm into the extracellular space. It is widely assumed that such bulk flow of bleeding cytoplasm takes WBs from the intact cell into the septal pore (Jedd & Chua, 2000; Markham & Collinge, 1987; Maruyama et al., 2005), thereby restricting the loss of cytoplasm largely to the injured cell (Jedd & Pieuchot, 2012). Here, we show that wound‐induced cytoplasmic bleeding in *Z. tritici* results in a drastic drop of pressure, indicated by bending of the adjacent septa towards the collapsed wounded cell and the occasional “ballooning” of the plasma membrane, which rapidly resealed over the wounded septal pore. Thus, we consider it likely that cytoplasmic bleeding from the intact cell could sweep WBs into the septal pore. It was reported that septa are sealed by a single WB (Markham & Collinge, 1987, this study) while the others are barely changing their position relative to the pore. Indeed, our 3D reconstruction of serial sections reveals only a small and insignificant shift of nonplugging WBs towards the septal pore (Student's *t* test; *p* = 0.6978). One may argue that a cytoplasmic bulk flow mechanism should reposition all WBs. The fact that this is not found was taken as an argument against a passive, bulk flow‐driven movement of WBs (Markham & Collinge, 1987). Unless a passive WB sealing mechanism is highly efficient, these results argue for a more active mechanism of WB translocation.

Although the majority of septal pores is sealed off from intact cells, we also find that WBs move from the ruptured cell into the septum. This raises more doubt about a passive cytoplasmic bulk flow‐driven mechanism, as WBs move against the cytoplasmic bleeding. Live cell observation of WBs shows that this movement occurs within ~1 s after cell injury, suggesting that it is mediated by force‐generating mechanisms. Active transport processes along the cytoskeleton spatially to organise the fungal cell (Lin et al., 2016), and it was suggested that microtubules are involved in WB motility into the septal pore (Markham & Collinge, 1987). To test this possibility, we performed laser‐rupture experiments in the presence of inhibitors that prevent formation of F‐actin or microtubules. However, we found no evidence for an involvement of the cytoskeleton in WB‐based closure of septal pores.

We found that WB movement into the septal pore of unwounded cells is induced when cellular ATP levels are depleted. This raises the possibility that chemical energy, or at least the presence of ATP, is required to keep the septal pore open. At present, the exact way by which ATP prevents WB activation is unclear. WBs are tethered to septa via Lah proteins (Beck et al., 2013; Han et al., 2014; Leonhardt et al., 2017), which show sequence similarity with the contractile muscle protein titin (Ng et al., 2009). Upon conformational change, titin can generate force (Martonfalvi, Bianco, Naftz, Ferenczy, & Kellermayer, 2017); this activity involves ATP binding to a kinase domain within titin (Puchner et al., 2008). Lah proteins lack such kinase domain, but our finding that cellular ATP is required to keep septal pores open suggests that as yet unknown kinases control WB movement into the pore. Indeed, studies in *A. nidulans* have shown that the NIMA kinase is involved in selective closure of the septal pore, yet this level of control occurs independently of WBs (Shen, Osmani, Govindaraghavan, & Osmani, 2014). Alternatively, ATP may bind directly to Lah or to interacting proteins, thereby affecting their activity. Such mechanisms have been well documented for a wide variety of membrane proteins and molecular chaperones (Suzuki & Yura, 2016; Wellhauser, Luna‐Chavez, D'Antonio, Tainer, & Bear, 2011). Although the detailed mechanism of WB translocation at low ATP levels is not known, it appears to be an efficient mechanism to ensure that cell rupture, and associated ATP depletion, results in rapid septal pore closure by WBs.

In conclusion, our results support a combinatorial mechanism for pore sealing by WBs. Although cytoplasmic bulk flow may be the primary way to close a pore, active recruitment of WBs for the ruptured cell may provide an alternative mechanism. The latter process appears to be ATP sensitive, and we speculate that it involves conformational changes in the Lah protein. Such an ATP‐dependent mechanism may also facilitate WB‐based closure of septal pores in intact cells (Bleichrodt et al., 2012; Markham, Collinge, Head, & Poole, 1987). Reversible closing of pores by WBs was suggested to underpin hyphal heterogeneity and cell specialisation (Bleichrodt et al., 2012).

## 4 EXPERIMENTAL PROCEDURES

### 4.1 Fungal strains and growth conditions

The *Z. tritici* wild‐type isolate IPO323 (Goodwin et al., 2011) was used to generate the strains IPO323_CHex1eGFP and IPO323_CHex1eGFP_HmCherrySso1, as well as strains IPO323_CeGFP (Kilaru, Schuster, Latz, et al., 2015) and IPO323_ GFPSso1 (Kilaru et al., 2017). All strains were grown in 20 ml YG media (yeast extract, 10 g/L; glucose, 30 g/L) at 18°C with 200 rpm for 48 hr.

### 4.2 Identification of ZtHex1 and bioinformatics

To identify homologues of WB‐associated proteins, we screened the published sequence of *Z. tritici* (http://genome.jgi.doe.gov/Mycgr3/Mycgr3.home.html), using the provided BLASTP function and the N. crassa protein sequences of HEX1 (NCBI reference: XP_963707.1). Sequences were obtained from the NCBI server (http://www.ncbi.nlm.nih.gov/pubmed). Sequence comparison was done using EMBOSS Needle (http://www.ebi.ac.uk/Tools/psa/emboss_needle/), and domain structures were analysed in PFAM (http://pfam.xfam.org/search/sequence; Finn et al., 2016). Phylogenetic trees were generated in MEGA5.2, using a maximum‐likelihood algorithm and 500 bootstrap cycles (http://www.megasoftware.net/; Tamura et al., 2011).

### 4.3 Molecular cloning

Vector pCHex1eGFP was designed for integration into the succinate dehydrogenase locus (Kilaru, Schuster, Latz, et al., 2015). It contains the gene for GFP, *egfp*, fused to gene*Zthex1*, placed under the control of constitutive *Zttub2* promoter and limited by the *Zttub2* terminator (Kilaru, Schuster, Latz, et al., 2015). In detail, plasmid pCHex1eGFP carries a 12,530‐bp fragment of pCeGFPTub2 (Schuster et al., 2015; digested with *Bsr*GI), a 1149‐bp *Z. tritici* α‐tubulin promoter (amplified with SK‐Sep‐14 and SK‐Sep‐47; Table 2), a 663‐bp full‐length *Zthex1* gene without stop codon (amplified with SK‐Sep‐156 and SK‐Sep‐157; Table 2), and a 717‐bp fragment, containing *egfp* (amplified with SK‐Sep‐16 and SK‐Sep‐78; Table 2). The vector was generated by in vivo ligation of these DNA fragments in the yeast Saccharomyces cerevisiae (Kilaru & Steinberg, 2015). Subsequent transformation into IPO323 was done as previously described (Kilaru, Schuster, Latz, et al., 2015), resulting in strain IPO323_CHex1eGFP. To covisualise WBs and the plasma membrane, vector pHmCherrySso1 (Kilaru et al., 2017) was ectopically integrated into IPO323_CHex1eGFP, resulting in strain IPO323_CHex1eGFP_HmCherrySso1.

**Table 2:** Primers used in this study

Primer name	Direction	Sequence (5′ to 3′)^a^
SK‐Sep‐14	Sense	*CATTTGCGGCTGTCTCGAAATCGACGGAAG*GCAGTCGACGCCAGATGATGG
SK‐Sep‐16	Sense	ATGGTGAGCAAGGGCGAGGAG
SK‐Sep‐47	Antisense	GGCGATGGTGGTATGCGGATG
SK‐Sep‐78	Antisense	*CCACAAGATCCTGTCCTCGTCCGTCGTCGC*TTACTTGTACAGCTCGTCCATGC
SK‐Sep‐156	Sense	*CATCACTCACATCCGCATACCACCATCGCC*ATGGGATATTACGACGAAGACGG
SK‐Sep‐157	Antisense	*GGTGAACAGCTCCTCGCCCTTGCTCACCAT*CAAGCGGCTACCGTGGACGAC

a Italics indicates part of the primer that is complementary with another DNA fragment to be ligated by homologous recombination in Saccharomyces cerevisiae.

### 4.4 Laser‐based epifluorescence microscopy

Fluorescence microscopy was performed as previously described (Kilaru, Schuster, Studholme, et al., 2015). In brief, the cells were inoculated in YG media (yeast extract, 10 g/L; glucose, 30 g/L) and grown at 18°C with 200 rpm for 48 hr and placed onto a 2% agar cushion and directly observed using a Olympus IX81 motorised inverted microscope (IX81; Olympus, Hamburg, Germany), equipped with a PlanApo 100×/1.45 Oil TIRF (Olympus, Hamburg, Germany). GFP‐labelled Hex1 and GFP or mCherry‐labelled SSO1 were excited using a VS‐LMS4 Laser Merge System with solid‐state lasers (488 nm/75 mW and 561 nm/75 mW; Visitron Systems, Puchheim, Germany). Simultaneous observation of red and green fluorescence was performed using a dual beam splitter (Dual‐View 2 Multichannel Imaging System; Photometrics, Tucson, USA), which was equipped with a dual‐line beam splitter (z491/561; Chroma Technology Corp., Bellows Falls, USA) with an emission beam splitter (565 DCXR; Chroma Technology Corp., Bellows Falls, USA), an ET bandpass 525/50 (Chroma Technology Corp., Bellows Falls, USA), and a single bandpass filter (BrightLine HC 617/73; Semrock, New York, USA). Z stacks were generated by using an objective piezo (Piezosystem Jena GmbH, Jena, Germany). Images were captured using a CoolSNAP HQ2 camera (Photometrics/Roper Scientific, Tucson, USA). All parts of the system were under the control of the software package MetaMorph (Molecular Devices, Wokingham, UK).

### 4.5 Laser‐induced rupture

Cells were grown in YG media (yeast extract, 10 g/L; glucose, 30 g/L) at 18°C with 200 rpm for 48 hr. One microlitre of the cell suspension was placed onto a 2% agar cushion and limitedly placed onto the microscope. Laser‐induced rupture was by a 200‐ms laser pulse, using a point‐focused 405 nm/60 mW diode laser. The laser was coupled into the light path by an OSI‐IX 71 adaptor (Visitron System, Puchheim, Germany) that was controlled by a UGA‐40 controller (Rapp OptoElectronic GmbH, Hamburg, Germany) and controlled by VisiFRAP 2D FRAP control software (Visitron System, Puchheim, Germany). The consequences of laser‐induced wounding were monitored in image sequences, acquired at an exposure time of 100–150 ms.

### 4.6 Drug treatments

Microtubules were depolymerised in strain IPO323_CHex1eGFP_HmCherrySso1 by incubating the cells in YG media containing 300 μM benomyl (Sigma‐Aldrich Gillingham, UK) for 30 min at 18°C with 200 rpm. The actin cytoskeleton was disrupted by treatment with 10 μM latrunculin A (Molecular Probes/Invitrogen, Paisley, UK) for 30 min at 18°C with 200 rpm. To disrupt microtubules and F‐actin simultaneously, we incubated cells of IPO323_CHex1eGFP_HmCherrySso1 with 300 μM benomy and 10 μM latrunculin A for 30 min at 18°C with 200 rpm. To deplete ATP cells, we incubated strain IPO323_CHex1eGFP_HmCherrySso1 with 100 μM CCCP (Sigma‐Aldrich Gillingham, UK) or 0.1% sodium azide (Sigma‐Aldrich Gillingham, UK), for 10–15 min at room temperature. In all cases, these treatments were followed by placing drug‐treated cells onto a 2% agar cushion, containing the respective inhibitor, directly observed under the microscope.

### 4.7 Quartz sand treatment of Z. tritici cells

Cells of strain IPO323 were grown in YG media (yeast extract, 10 g/L; glucose, 30 g/L) at 18°C, 200 rpm for 48 hr. Cell suspension of 1 ml was mixed with ≈500 mg of acid‐washed glass beads (425–600 μm; Sigma‐Aldrich, Gillingham, UK) and mixed for 15 min in a 2‐ml reaction tube, using IKA Vibrax shaker (IKA, Staufen, Germany). Glass beads were removed by centrifugation at 1000 rpm for 30 s. The cell‐containing supernatant was prepared for electron microscopy as described below.

### 4.8 Ultrastructural studies

For ultrastructural studies, liquid cultures were fixed and embedded as previously described (Schuster et al., 2016). For serial section analysis, sections of 70 nm were placed on pioloform‐coated copper slot grids (Agar Scientific, Stansted, UK) and contrasted with lead citrate. Sections were examined using a JEOL JEM 1400 transmission electron microscope operated at 120 kV, and images taken with a digital camera (ES 100W CCD, Gatan, Abingdon, UK). Estimation of WB numbers at septal pores was done by the physical disector method (Sterio, 1984). To this end, 25 ± 1 sections were acquired at a magnification of 60,000 × g. WBs were identified by their characteristic appearance, excluding those that were still in contact with peroxisomes. The average distance of WBs to the septal pore was measured by defining the centre of the pore in Photoshop CS6 and the centre of WBs, taking *z*‐axis information into account. Three‐dimensional models of septum‐associated WBs were reconstructed from the serial micrographs using IMOD software and ETomo (http://bio3d.colorado.edu/imod/), and video files created with ImageJ (https://imagej.nih.gov/ij/).

## Supporting information


**Movie S1**. 3D‐reconstruction of an image stack, derived from serial sections, showing septum‐associate WBs in *Z. tritici*. The animation was generated using IMOD and ImageJ software (see main text; Experimental Procedures). Scale bar represents 500 nm.

**Movie S2**. Laser rupture of a hypha of *Z. tritici.* Three cells are separated from each other by septa (arrowheads in first frame). After a laser pulse (indicated by “LASER”), the cytoplasm of the injured cell, labelled with cytoplasmic eGFP bleeds into the extracellular space. The other cells are not affected. Time is given in seconds and milliseconds. The scale bar represents 10 μm.

**Movie S3**. Behaviour of septa, labelled with the plasma membrane marker eGFP‐Sso1 (Kilaru et al., 2017), after laser rupture. The injured cell collapses and the septum bends, indicating a pressure gradient between both cells. Time is given in seconds and milliseconds. The scale bar represents 2 μm.

**Movie S4**. Flow of cytoplasm, labelled with cytoplasmic GFP, after laser‐induced rupture of the lower cell (injured cell). While the cytoplasm bleeds out of the wounded cell, little cytoplasmic movement is seen in the intact cell, suggesting that WB sealing is an efficient process in *Z. tritici*. Location of septa is indicated by yellow arrowheads. Time is given in seconds and milliseconds. The scale bar represents 2 μm.

**Movie S5.** Motility of a cytoplasmic WB. Time is given in seconds and milliseconds. The scale bar represents 3 μm.

**Movie S6**. Movie shows the behaviour of WBs (labelled by ZtHex1‐eGFP) relative to the septum (labelled with the plasma membrane marker mCherry‐Sso1). After laser rupture of the lower cell, the septum bends towards the ruptured cell and a WB plugs the septal pore. This pressure gradient may flush the WB from the intact cell (right) into the septal pores. WBs in the ruptured cell remain associated with the septum. Time is given in seconds and milliseconds. The scale bar represents 2 μm.

**Movie S7**. Movie shows WBs, labelled by ZtHex1‐eGFP, after laser rupture of the left cell. While WBs from the intact cell close the septal pore, WBs in the ruptured cell remain associated with the septum via a flexible linker. Numbers indicate different cells. Time is given in seconds and milliseconds. The scale bar represents 1 μm.

**Movie S8**. Movie shows dynamic rearrangement of ZtHex1‐GFP after rupture of the left cell using a laser pulse. Example #2 and #4 shows “ballooning”, which is due to the extension of the plasma membrane (see Movie 8). Numbers indicate independent experiments. Time indicates seconds and milliseconds after wounding; scale bar represents 1 μm.

**Movie S9**. Movie shows “ballooning” of ZtHex1 and the plasma membrane syntaxin ZtSso1 after laser injury of a cell. Note that the syntaxin ZtSso1 contains a transmembrane domain and, therefore, identifies the “balloon” as being plasma membrane derived. Time indicates seconds and milliseconds after wounding; scale bar represents 1 μm.

**Movie S10**. Movie shows recruitment of WBs from the ruptured cell into the septal pore. Time indicates seconds and milliseconds after wounding; scale bar represents 1 μm.

**Figure S1.** Electron micrographs of septal pores in wild‐type cells of strain IPO323 after wounding with quartz sand. WBs seal the septal pore on the side of the ruptured cell (indicated by “Dead cell”). This situation represents a minority of all observed cases (~15%). Scale bar represents 0.2 μm
