# Supplementary material for: ATP prevents Woronin bodies from sealing septal pores in unwounded cells of the fungus Zymoseptoria tritici
Source: Cell Microbiol. 2017 Aug 9;19(11):e12764. doi: 10.1111/cmi.12764 (PMC5656841; doi:10.1111/cmi.12764)
Supplement: Supplementary file 11 — Figure S1. Electron micrographs of septal pores in wild‐type cells of strain IPO323 after wounding with quartz sand. WBs seal the septal pore on the side of the ruptured cell (indicated by “Dead cell”). This situation represents a minority of all observed cases (~15%). Scale bar represents 0.2 μm [file CMI-19-na-s011.docx]

**Supplementary Figures and Figure legends**

**
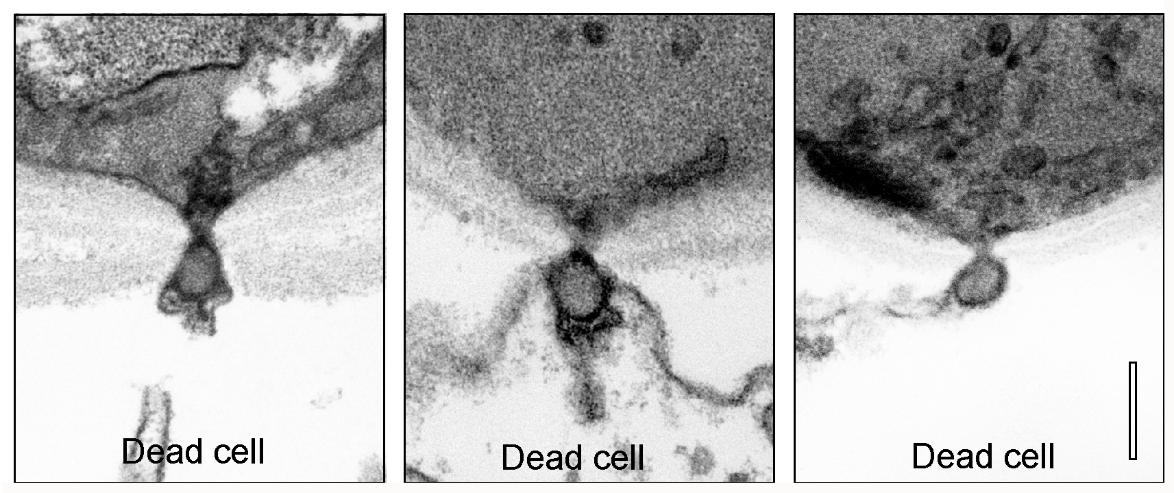
**

**Figure S1.** Electron micrographs of septal pores in wild-type cells of strain IPO323 after wounding with quartz sand. WBs seal the septal pore on the side of the ruptured cell (indicated by “Dead cell”). This situation represents a minority of all observed cases (~15%). Scale bar represents 0.2 µm

**Supplementary movie legends**

**Movie 1**. 3D-reconstruction of an image stack, derived from serial sections, showing septum-associate WBs in *Z. tritici*. The animation was generated using IMOD and ImageJ software (see main text; Experimental Procedures). Scale bar represents 500 nm.

**Movie 2**. Laser rupture of a hypha of *Z. tritici.* Three cells are separated from each other by septa (arrowheads in first frame). After a laser pulse (indicated by “LASER”), the cytoplasm of the injured cell, labelled with cytoplasmic eGFP bleeds into the extracellular space. The other cells are not affected. Time is given in seconds and milliseconds. The scale bar represents 10 µm.

**Movie 3**. Behaviour of septa, labelled with the plasma membrane marker eGFP-Sso1 (Kilaru et al, 2017), after laser rupture. The injured cell collapses and the septum bends, indicating a pressure gradient between both cells. Time is given in seconds and milliseconds. The scale bar represents 2 µm.

**Movie 4**. Flow of cytoplasm, labelled with cytoplasmic GFP, after laser-induced rupture of the lower cell (injured cell). While the cytoplasm bleeds out of the wounded cell, little cytoplasmic movement is seen in the intact cell, suggesting that WB sealing is an efficient process in *Z. tritici*. Location of septa is indicated by yellow arrowheads. Time is given in seconds and milliseconds. The scale bar represents 2 µm.

**Movie 5.** Motility of a cytoplasmic WB. Time is given in seconds and milliseconds. The scale bar represents 3 µm.

**Movie 6**. Movie shows the behaviour of WBs (labelled by ZtHex1-eGFP) relative to the septum (labelled with the plasma membrane marker mCherry-Sso1). After laser rupture of the lower cell, the septum bends towards the ruptured cell and a WB plugs the septal pore. This pressure gradient may flush the WB from the intact cell (right) into the septal pores. WBs in the ruptured cell remain associated with the septum. Time is given in seconds and milliseconds. The scale bar represents 2 µm.

**Movie 7**. Movie shows WBs, labelled by ZtHex1-eGFP, after laser rupture of the left cell. While WBs from the intact cell close the septal pore, WBs in the ruptured cell remain associated with the septum via a flexible linker. Numbers indicate different cells. Time is given in seconds and milliseconds. The scale bar represents 1 µm.

**Movie 8**. Movie shows dynamic rearrangement of ZtHex1-GFP after rupture of the left cell using a laser pulse. Example #2 and #4 shows “ballooning”, which is due to the extension of the plasma membrane (see Movie 8). Numbers indicate independent experiments. Time indicates seconds and milliseconds after wounding; scale bar represents 1 µm.

**Movie 9**. Movie shows “ballooning “of ZtHex1 and the plasma membrane syntaxin ZtSso1 after laser injury of a cell. Note that the syntaxin ZtSso1 contains a transmembrane domain and, therefore, identifies the “balloon” as being plasma membrane derived. Time indicates seconds and milliseconds after wounding; scale bar represents 1 µm.

**Movie 10**. Movie shows recruitment of WBs from the ruptured cell into the septal pore. Time indicates seconds and milliseconds after wounding; scale bar represents 1 µm.
